# Large-scale brain network associated with creative insight: combined voxel-based morphometry and resting-state functional connectivity analyses

**DOI:** 10.1038/s41598-018-24981-0

**Published:** 2018-04-24

**Authors:** Takeshi Ogawa, Takatsugu Aihara, Takeaki Shimokawa, Okito Yamashita

**Affiliations:** 1 Advanced Telecommunication Research Institute International, Cognitive Mechanisms Laboratories, Kyoto, 619-0288 Japan; 2 Advanced Telecommunication Research Institute International, Neural Information Analysis Laboratories, Kyoto, 619-0288 Japan

## Abstract

Creative insight occurs with an “Aha!” experience when solving a difficult problem. Here, we investigated large-scale networks associated with insight problem solving. We recruited 232 healthy participants aged 21–69 years old. Participants completed a magnetic resonance imaging study (MRI; structural imaging and a 10 min resting-state functional MRI) and an insight test battery (ITB) consisting of written questionnaires (matchstick arithmetic task, remote associates test, and insight problem solving task). To identify the resting-state functional connectivity (RSFC) associated with individual creative insight, we conducted an exploratory voxel-based morphometry (VBM)-constrained RSFC analysis. We identified positive correlations between ITB score and grey matter volume (GMV) in the right insula and middle cingulate cortex/precuneus, and a negative correlation between ITB score and GMV in the left cerebellum crus 1 and right supplementary motor area. We applied seed-based RSFC analysis to whole brain voxels using the seeds obtained from the VBM and identified insight-positive/negative connections, i.e. a positive/negative correlation between the ITB score and individual RSFCs between two brain regions. Insight-specific connections included motor-related regions whereas creative-common connections included a default mode network. Our results indicate that creative insight requires a coupling of multiple networks, such as the default mode, semantic and cerebral-cerebellum networks.

## Introduction

Creativity is a crucial cognitive function to achieve innovation and civilised daily living. Creativity enables humans to solve problems by generating novel and useful products or ideas. Recently, creative cognition was divided into the three following categories: domain-general creative thought, domain-specific creative thought, and creative insight^1–3^. Domain-general creative thought is predominantly measured using divergent thinking task paradigms (e.g. the Alternate Uses task^4^, Torrance Test of Creative Thinking^5^ and S-A Creativity test^6,7^), in which participants are instructed to generate several possible solutions to an open-ended problem. Domain-specific creative thought is assessed via artistic performance in domains such as music^8–10^, literature^11^ and visual arts^12,13^. In contrast to both domain-general and domain-specific creative thought, creative insight requires participants to choose particular answers from many possibilities. Creative insight can be measured using verbal (e.g. the remote associates test: RAT), mathematical (e.g. matchstick arithmetic task: MA) and spatial (e.g. nine-dot problem) insight problem tasks are used to measure the performance of creative insight^14,15^.

Beaty *et al*. suggested that processes of creative cognition comprise the following two components: idea generation and evaluation^1^. The process of idea generation is needed to diffuse attention and to find possible answers in a bottom-up manner, whereas the process of idea evaluation involves focused attention and cognitive control. For example, divergent thinking requires a higher level of idea generation to produce many items, but a lower level of idea evaluation because of a weakly constrained process. In contrast to idea generation, the process of idea evaluation identifies the appropriateness of a correct answer or helpful solution to a difficult problem^14,15^. Creative insight requires processes of both idea generation and idea evaluation. Integration of several large-scale networks may therefore be required to achieve creative cognition.

Recent neuroimaging studies have examined a large-scale brain network associated with divergent thinking^4,5,7^. The process of divergent thinking spontaneously involves high idea generation with weakly constrained conditions. In the large-scale brain network, the function of the default mode network (DMN) represents individual performance correlated with working memory tasks^7^ and financial decision-making tasks^16^. Both morphological^6,17–19^ and functional^4,7,20,21^ imaging studies have indicated that the precuneus, a hub of the DMN, is engaged in divergent thinking. In addition, studies of creative cognition suggest that spontaneous brain activity is involved in self-generated thought, such as mind wandering, future thinking, perspective taking and mental simulation^1^.

However, insight problem solving is strongly constrained by task demands and involves not only idea generation but also idea evaluation to identify a particular solution from many possibilities to a difficult problem^22^. Jung-Beeman *et al*.^14^ summarised four characteristics of insight as follows: (i) solvers first come to an impasse, no longer progressing towards a solution, (ii) solvers usually cannot report the processing that enables them to reinterpret the problem and overcome the impasse, (iii) solvers experience their solutions as arising suddenly and immediately recognise the correctness of the solution (or solution path), (iv) performance on insight problems is associated with creative thinking and other cognitive abilities that are different from those associated with performance on non-insight problems. Functional magnetic resonance imaging (fMRI) and electroencephalography (EEG) have been used to measure brain activity during a verbal insight problem task to test the empirically and theoretically derived hypothesis that solving problems with insight requires engagement of distinct neural mechanisms in the right hemisphere anterior temporal lobe^14^. Jung-Beeman *et al*. demonstrated that the anterior temporal lobe exhibited greater activity for an insight solution than for a non-insight solution. In addition, EEG alpha/gamma band activity increased (predominantly in the right posterior cingulate cortex (PCC)/superior temporal gyrus (STG)) just before insight solution. Using another approach to investigate the neural mechanisms of insight problem solving, Chi and Snyder used transcranial direct current stimulation to facilitate behavioural performance of MA^15^. Although previous neuroimaging and brain stimulation findings have demonstrated correlational and causal relationships between insight solution and specific brain areas, a large-scale network underlying insight-specific processes has not been investigated.

The identification of a large-scale network associated with creative cognition has been explored on the basis of a strong hypothesis. Beaty *et al*. defined seeds within the DMN and the executive control network that were implicated in divergent thinking, as revealed by multivariate pattern analysis^4^. Chen *et al*.^23^ and Takeuchi *et al*.^7,24^ defined seeds based on the Montreal Neurological Institute (MNI) coordinates of previous studies. However, the association between creative insight and a large-scale network is poorly understood. Therefore, it is difficult to apply resting-state functional connectivity (RSFC) analyses based on seed regions associated with creative insight.

In this study, we aimed to understand large-scale brain networks associated with creative insight. A large-scale brain network associated with creative insight could be represented by performance on insight tasks, not only anatomically but also functionally, without a strong hypothesis. A total of 232 participants aged 21 to 69 years completed an insight task battery (ITB) consisting of three insight tasks (matchstick arithmetic task: MA^25^, remote associates test: RAT^26^ and insight problem solving task: IP^27^) and a 10 min resting-state fMRI scan. To identify brain regions associated with the ITB, we applied voxel-based morphometry (VBM) analysis of anatomical MRI data to ITB scores. We conducted a multiple regression analysis based on seed-based RSFC results using the brain regions extracted from the VBM analysis as seeds. We not only identified common networks involved in creative cognition, but also insight-specific networks from the integrated anatomical and functional information.

## Results

### Behavioural data

A total of 232 participants completed the ITB (see Supp. Fig. 1). We excluded 70 participants who had experienced MA or IP prior to this experiment; data from a final total of 162 participants were included in the VBM analysis. Table 1 shows the numbers of participants for each age range and sex.

**Table 1:** Number of participants within each age range in the VBM analysis.

Age range (years)	20–29	30–39	40–49	50–59	60–69	Total
Male *n*	19	20	19	13	17	88
Female *n*	7	18	17	18	14	74
Total *n*	26	38	36	31	31	162

**Figure 1:**
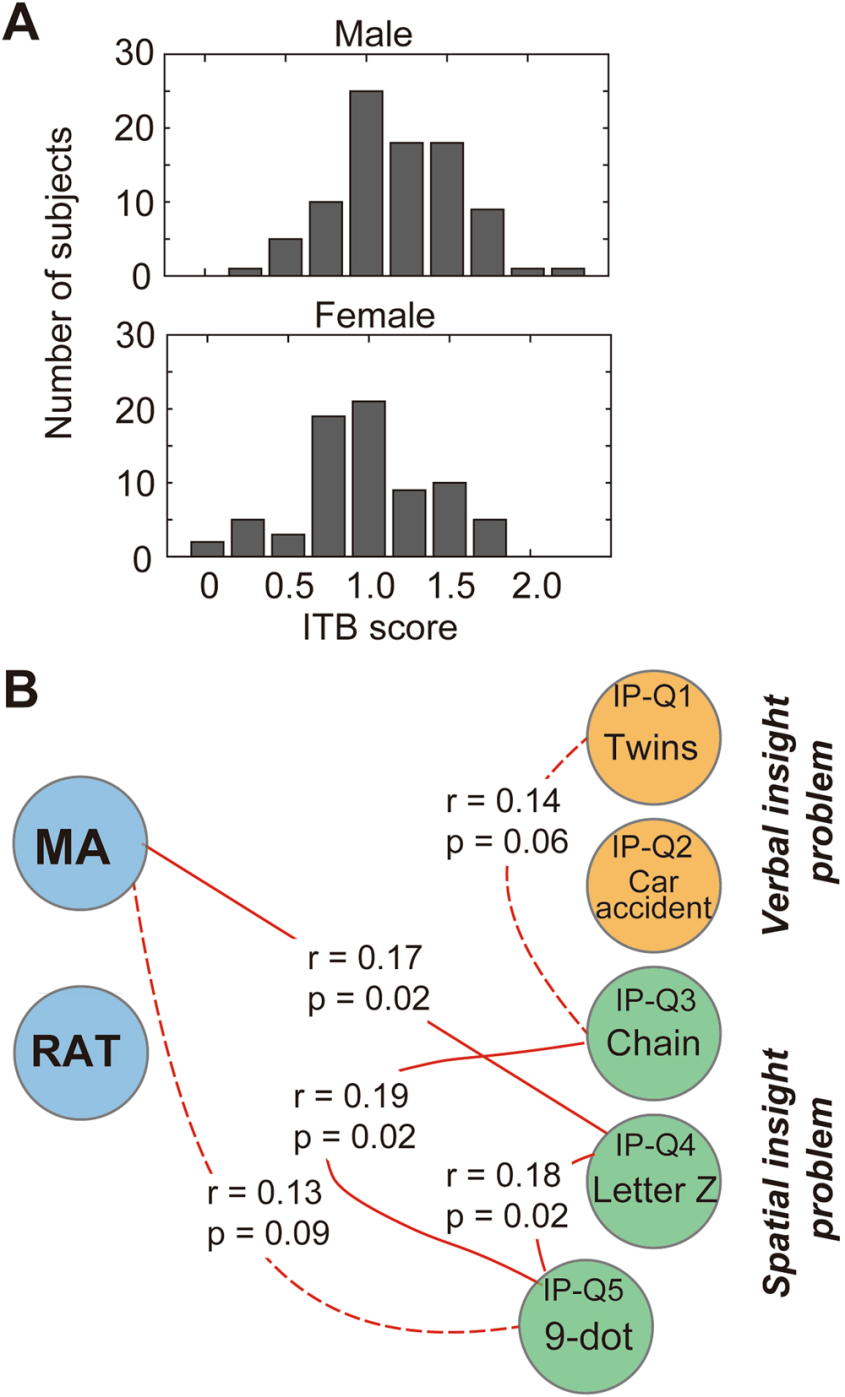
Distribution of the ITB score and the relationship between MA, RAT and IP scores. (**A**) Histogram of the ITB scores of male (upper panel) and female (bottom panel) participants, which were significantly different (one-way ANOVA, *p* = 0.011). (**B**) Results of the partial correlation analysis of the MA, RAT and IP results, controlling for age, sex and cohort group (01/02). Red lines indicate a positive correlation (solid line: *p* < 0.05, dotted line: *p* < 0.1). Only correlations for which *p*-values were less than 0.1 are shown. For the IP, we selected five questions (twins, car accident, chain, letter Z and the 9-dot problem; see the previous study of Dow and Mayer^27^). Abbreviations: IP: insight problem task; ITB: insight test battery; MA: matchstick arithmetic task; RAT: remote associates test.

MA, RAT and IP results are illustrated in Fig. 1 and Table 2. We normalised each task score so that scores ranged from 0 to 1, and summed them as the ITB score. Mean (±SD) accuracies of the MA, RAT and IP scores were 0.44 ± 0.21, 0.45 ± 0.20 and 0.31 ± 0.21, respectively. The ITB scores followed a normal distribution with a mean ± SD of 1.20 ± 0.41, and there was a significant difference between male and female participants (one-way ANOVA; *F* = 11.03, *p* = 0.0011, Fig. 1A). Table 2 shows the mean accuracies of all questions. We assumed that the MA and RAT were simplified tasks of the IP and therefore evaluated the relationship between MA, RAT and IP scores using a partial correlation analysis (Fig. 1B). The MA score was positively correlated with IP-Q4 (*r* = 0.17, *p* = 0.02) and IP-Q5 (*r* = 0.13, *p* = 0.09). IP-Q1 was positively correlated with IP-Q3 (*r* = 0.14, *p* = 0.06). IP-Q5 was positively correlated with IP-Q3 (*r* = 0.19, *p* = 0.02) and IP-Q4 (*r* = 0.18, *p* = 0.02). In this correlation analysis, there were no correlations with the IP scores that were common to MA and RAT scores, but the MA score was associated with spatial insight problems.

**Table 2:** Mean accuracy for the creative insight task.

*Matchstick Arithmetic Task (MA)*	Correct rate
Q1 (Type I)	0.78
Q2 (Type I)	0.22
Q3 (Type I)	0.76
Q4 (Type II)	0.01
Mean accuracy ± SD	0.44 ± 0.21
***Remote Associates Test (RAT)***	**Correct rate**
Q1	0.89
Q2	0.73
Q3	0.24
Q4	0.43
Q5	0.58
Q6	0.59
Q7	0.46
Q8	0.35
Q9	0.07
Q10	0.13
Mean accuracy ± SD	0.45 ± 0.20
***Insight Problem Task (IP)***	**Correct rate**
Q1 (twins)	0.52
Q2 (car accident)	0.49
Q3 (chain)	0.17
Q4 (letter Z)	0.24
Q5 (the 9 dots)	0.15
Mean accuracy ± SD	0.31 ± 0.21
Mean accuracy ± SD of total score	1.20 ± 0.41

### Correlation between grey matter volume and the insight score

**Figure 2:**
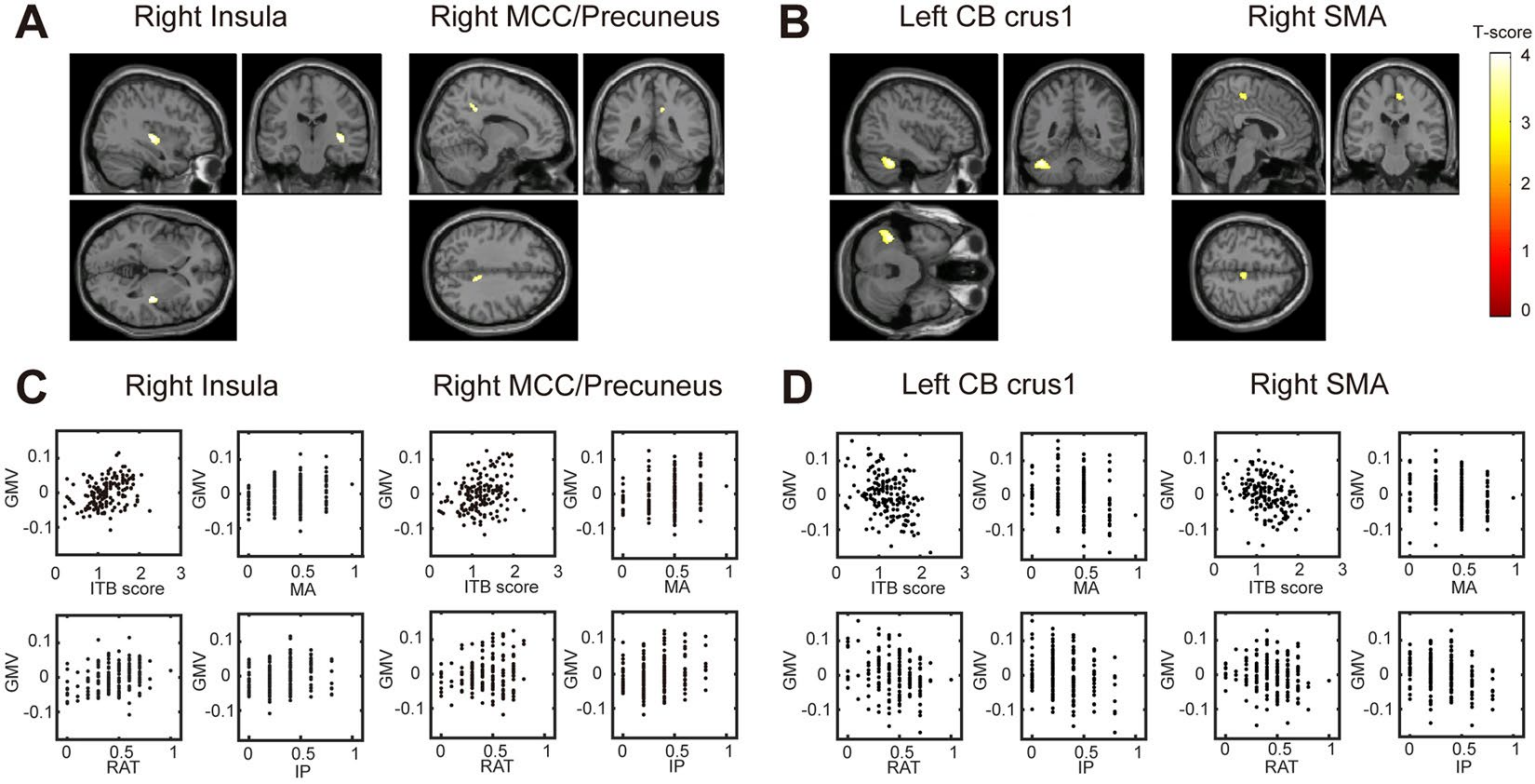
Brain regions with positive correlation between GMV and ITB score. (**A**) Regions showing positive correlation between GMV and ITB score (*p* < 0.001, uncorrected). Regions showing significant correlation are overlaid on a single subject T1 image of SPM12. Significant correlations were found in the right insula and right MCC/precuneus. (**B**) A negative correlation between GMV and ITB score was found in the left CB crus 1 and the right SMA. (**C**) Scatter plots of the relationship between each score (ITB, MA, RAT and IP) and GMV in the right insula and right MCC/precuneus. (**D**) Scatter plots of the relationship between each score and GMV in the left CB crus 1 and right SMA. Abbreviations: CB: cerebellum; GMV: grey matter volume; IP: insight problem task; ITB: insight test battery; MA: matchstick arithmetic task; MCC: middle cingulate cortex; RAT: remote associates test; SMA: supplementary motor area.

We investigated the association between grey matter volume (GMV) and the ITB score after controlling for age, sex (male/female), MRI scanner (Trio/other, Prisma/other), cohort (group 01/group 02) and the total brain volume (TBV: sum of GMV and white matter volume) of individual brains using multiple linear regression analysis (see Methods) in SPM12 (Wellcome Trust Centre for Neuroimaging, London, UK; www.fil.ion.ucl.ac.uk/spm/). Table 3 presents statistical information on the brain regions in the extracted cluster in which there was a correlation between GMV and the ITB score. The results revealed that the ITB score was positively correlated with GMV (Fig. 2A,B) centred at the right insula (x, y, z = 40, −13, 4; *t* score = 4.04, *p(uncorr)* < 0.001) and right middle cingulate cortex (MCC)/precuneus (x, y, z = −19, −51, 43, *t* score = 3.67, *p(uncorr)* < 0.001), labelled by the Anatomical Automatic Labelling 2 (AAL2) toolbox. In addition, we found two clusters with GMVs that were negatively correlated with the ITB score in the left cerebellum crus 1 (CB crus 1; x, y, z = −40, −48, −32, *t* score = −4.44, *p(uncorr)* < 0.001), and the right supplementary motor area (SMA; x, y, z = 6, −24, 52, *t* score = −3.76, *p(uncorr)* < 0.001).

**Table 3:** MNI coordinates of peak voxels in each cluster identified by VBM analysis.

Brain regions	Peak coordinate (MNI)			Cluster size		Details (%)
	X	Y	Z	voxels	Peak of T score	
Right Insula	40	−14	−4	347	4.04	R insula: 53.31; OUTSIDE: 34.87 R STG: 9.22; R putamen: 2.59
Right MCC/Precuneus	14	−39	36	138	3.67	R MCC: 57.25; R precuneus: 40.58 OUTSIDE: 2.17
Left CB Crus1	−40	−48	−32	749	−4.44	L CB Crus 1: 81.98 L CB 6: 11.62 OUTSIDE: 5.61 L ITG: 0.40 L Fusiform: 0.40
Right SMA	6	−24	52	121	−3.76	R SMA: 92.56 R MCC: 4.96 R Para Central L: 2.48

Four clusters were identified by the correlation analysis (*p* < 0.001, uncorrected, cluster size >112 voxels). Co-localisation of brain regions and their volumes were obtained using the AAL2 toolbox.

Abbreviations: CB: cerebellum; ITG: inferior temporal gyrus; L: left; MCC: middle cingulate cortex; R: right; SMA: supplementary motor area; STG: superior Temporal Gyrus.

To confirm the correlation profiles for each task (MA, RAT and IP), we plotted the relationship between GMV and each task score at the peak voxel of each cluster across participants (Fig. 2C,D). We calculated Pearson’s correlation coefficients from the right insula, right MCC/precuneus, left CB crus 1 and right SMA, respectively. We found significant correlations between task scores and GMV in the right insula (MA: *r* = 0.20, *p* = 0.01; RAT: *r* = 0.28, *p* = 2e-4; IP: *r* = 0.17, *p* = 0.02) as well as a positive correlation between the ITB score and GMV (*r* = 0.39, *p* = 1e-6). In the right MCC/precuneus, the relationships between each of the three tasks and GMV also tended to be positively correlated with GMV (MA: *r* = 0.29, *p* = 1e-4; RAT: *r* = 0.15, *p* = 0.05; IP: *r* = 0.21, *p* = 6e-3) in addition to a positive correlation between the ITB score and GMV (*r* = 0.34, *p* = 9e-7) (Fig. 2C). In the left CB crus 1, scores of each of the three tasks were negatively correlated with GMV (MA: *r* = −0.208, *p* = 0.007; RAT: *r* = −0.273, *p* = 0.0004; IP: *r* = −0.232, *p* = 0.003). In the right SMA, scores of each of the three tasks were also negatively correlated with GMV (MA: *r* = −0.222, *p* = 0.004; RAT: *r* = −0.135, *p* = 0.09; IP: *r* = −0.255, *p* = 0.001) (Fig. 2D). These results suggest that the brain regions identified by the VBM analysis were significantly correlated with not only the ITB score but also each task score, although significance levels between the task score and GMV were different.

### Association between the ITB score and the strength of RSFC

The data of all participants were preprocessed with a canonical resting-state fMRI analysis procedure (see Methods). To reduce spurious changes in RSFC by head motion, the data were evaluated with a method used for reducing motion-related artefacts in resting-state fMRI^28^. For the seed-based RSFC analysis, we selected four clusters as seeds (seed 1: right insula; seed 2: right MCC/precuneus; seed 3: left CB crus 1; seed 4: right SMA) according to the VBM analysis (Table 3). Subsequently, the time course of each seed was extracted by averaging across voxels within each cluster. We calculated individual RSFC correlation maps (*r*-maps) represented by the Pearson’s correlation coefficient between the time course of each seed and the time course for each of the other voxels, then we computed Fisher’s z-transform to improve the normality of the *r*-map.

**Figure 3:**
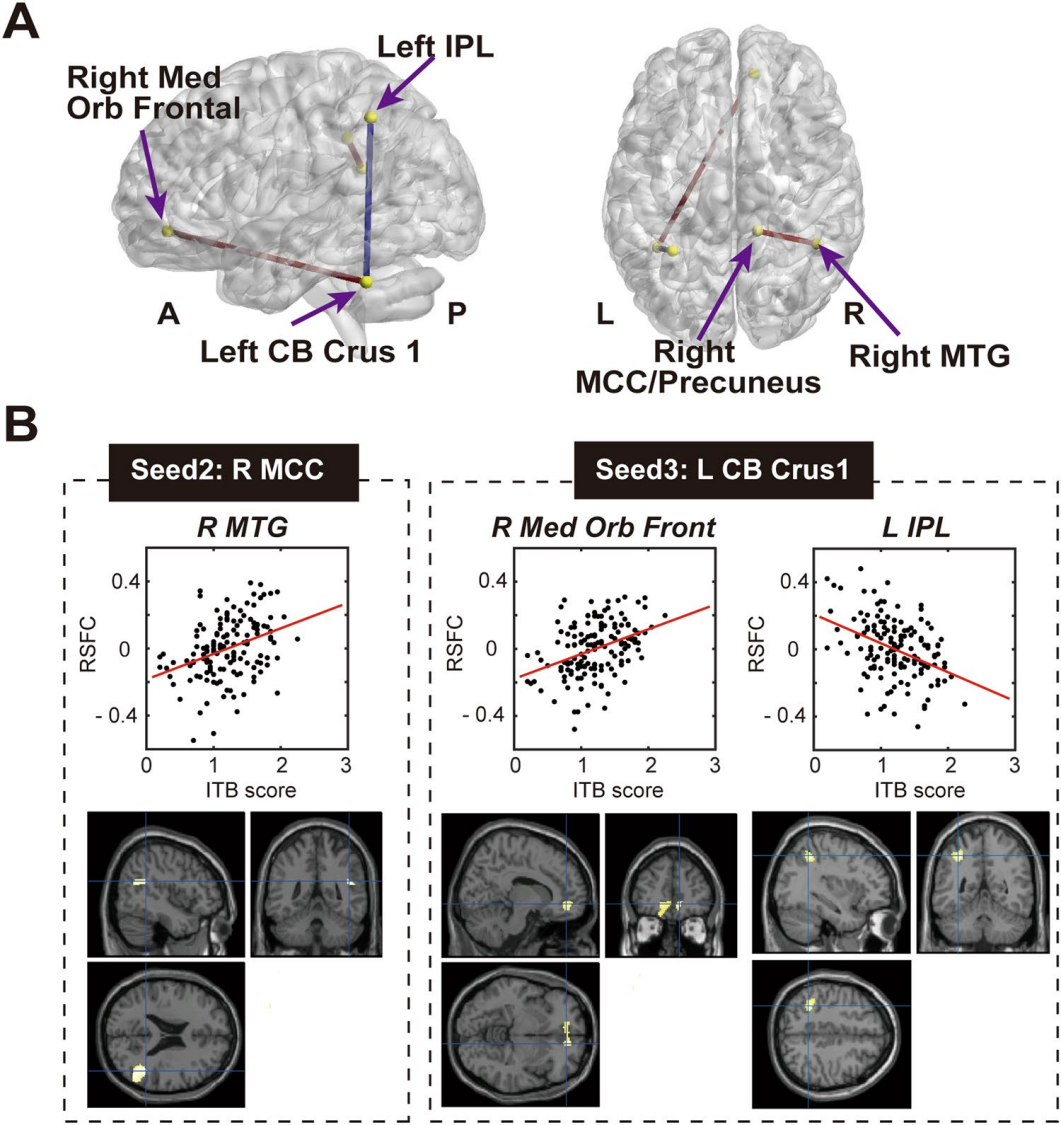
Large-scale network association between the strengths of RSFC and the ITB score. (**A**) Correlated clusters and seeds (yellow spheres) with a threshold of *p* < 0.001 uncorrected and cluster level FWE at *p* < 0.1 or *p* < 0.05. Connections between the seed to cluster at the peak of each cluster (blue lines show insight-negative connections and red lines show insight-positive connections). (**B**) The associations between individual ITB scores and individual RSFCs at the peak voxel from the seeds and their regions of significant correlation are overlaid on a single subject T1 image of SPM12. The Pearson’s correlations (*r*) and its *p* values are provided in Table S1. Abbreviations: CB: cerebellum; FWE: Family-Wise Error; IPL: inferior parietal lobule; MCC: middle cingulate cortex; MTG: middle temporal gyrus; ITB: insight test battery; Med Orbito Front: medial orbitofrontal; IPL: inferior parietal lobule; RSFC: resting-state functional connectivity.

In the second level analysis, we examined the relationship between the individual ITB score and the strength of RSFC for each seed. We applied multiple regression analysis to model a linear relationship between the ITB score and the strength of RSFC from each seed to whole voxels using SPM12. We found insight-positive/negative connections, which had significant positive/negative correlations between the individual ITB scores and the strength of RSFC (Fig. 3A). Table 4 shows information from extracted clusters (peak level < 0.001 uncorrected and cluster level Family-Wise Error, *p* < 0.1 or 0.05).

**Table 4:** MNI coordinates of peak voxels in each cluster identified by seed-based RSFC analysis.

Seed	Target brain regions (sub-regions with percentage)	Peak coordinate (MNI)				
		X	Y	Z	Cluster size	Peak of T score
Right MCC/Precuneus	*R MTG (R MTG: 38.5; R Angular gyrus: 34.1; R STG: 24.6; OUTSIDE: 2.4 R Middle occipital gyrus: 0.5)	46	−46	22	236	3.84
Left CB Crus1	**R Med Orb Frontal (R Medial orbitofrontal: 39.1; L Medial orbitofrontal: 25.2; L ACC: 12.9; L Medial orbital gyrus: 12.6; OUTSIDE: 3.6; R Superior frontal gyrus, medial: 0.3)	12	46	−6	304	4.21
	**L IPL (L IPL: 96.4; L SPL: 2.0; OUTSIDE: 1.6)	−32	−50	46	247	−4.47

Abbreviations: ACC: anterior cingulate cortex; IPL: inferior parietal lobule; L: left; MCC: middle cingulate cortex; MTG: middle temporal gyrus; R: right; SPL: superior parietal lobule; STG: superior temporal gyrus; peak level p < 0.001 uncorrected and cluster level Family-Wise Error *p < 0.1 or **p < 0.05.

We found two insight-positive connections (right MCC/precuneus-right middle temporal gyrus (MTG); left CB crus 1-right medial orbitofrontal cortex (Med Orb Front)), and one insight-negative connection (left CB crus 1-left inferior parietal lobule (IPL)). Individuals with higher creative insight showed higher RSFC between the right MCC/precuneus and right MTG, and between the left CB crus1 and right Med Orb Front, and lower of RSFC between the left CB crus1 and left IPL.

## Discussion

For the first time, we identified potential large-scale networks that underlie creative insight. We investigated GMV and RSFC associated with creative insight in participants across a wide age range using an exploratory approach. The VBM analysis revealed that GMV-increased clusters centred at the right insula and right MCC/precuneus, and GMV-decreased clusters centred at the left CB crus 1 and right SMA were associated with creative insight, as measured by the ITB score (MA, RAT and IP scores). To understand the relationship between individual creative insight and individual RSFC, we proceeded to seed-based RSFC analysis constrained by the results of the VBM analysis. We identified three connections (insight-positive connections: right MCC/precuneus-right MTG, left CB crus 1-right Med Orb Front; insight-negative connection: left CB crus 1-left IPL). The right MCC/precuneus is widely accepted as a key node in the DMN, and the CB crus 1 is associated with sensorimotor processing. Our results suggest that higher creative insight is achieved through not only creativity-common networks, including the DMN, but also insight-specific connections such as semantic and cerebral-cerebellum networks^13,14^. We will first discuss functions of the seed regions based on the results of VBM-constrained RSFC analysis. We will then discuss the functions of insight-positive/negative connections.

The GMV of a cluster extending from the right posterior MCC to the right precuneus was positively correlated with the ITB score. This is consistent with results of other studies in which creative cognition was assessed by a divergent thinking task^6,17^ and a creative achievement questionnaire (CAQ)^23^. The GMV of the precuneus and right subcortical regions that are part of the dopaminergic system have been found to be positively correlated with individual creativity measured by divergent thinking, such as the S-A creativity test^29^, which yields total creativity scores as well as scores within four dimensions (fluency, originality, elaboration and flexibility)^6^. In the case of the Alternate Uses task used to assess divergent thinking, the GMV in the right precuneus has been associated with creative potential, particularly, ideational originality^17^. Similarly, Chen *et al*. found a positive correlation between GMV in the left precuneus and the CAQ, which measures individual real-life creative performance involving divergent thinking and creative personality traits^23^. A higher GMV in the MCC/precuneus is associated with the process of higher idea generation required for divergent thinking and insight.

In addition, we found an insight-positive connection from the MCC/precuneus to the right MTG. The MTG plays key roles in language and associative-semantic processing^5,30,31^, as well as the restructuring process that is an essential property of insight problem solving^32^. The MTG might also be activated by integration or retrieval processes, especially language and associative-semantic processes. The functional connection between the precuneus and temporal regions may also be associated with increased demands on memory retrieval mechanisms during divergent thinking tasks. Therefore, coactivation in the MCC/precuneus and MTG may be necessary for idea evaluation and generation in creative cognition.

In the VBM analysis, we identified a cluster within the insula in which the GMV was positively correlated with the ITB score. According to previous reviews^33,34^, the insula is divided into three parts, anterior, dorsal and posterior regions. Klein *et al*. reported that functions of the insula are associated with cognitive control, decision making, emotion, autonomic interoception, and somatosensory perception^34^. In particular, the anterior insula is a major hub of the salience network in humans^35^ and macaques^36^. Previous fMRI studies of divergent thinking have reported that functional connectivity between the DMN, executive control network, and saliency network were increased during a verb generation task, which is a measure of the domain-general creativity^4,37^. However, we identified a cluster in the posterior insula using the VBM analysis, and did not find a significant RSFC network from this cluster. Two previous studies have described the brain structural feature of the posterior insula^38,39^. Zheng *et al*. found that GMV in the right posterior insula is positively correlated with individual modesty^38^. They suggested that modesty is related to justice and egalitarianism, and allows people to have an accurate perception of the self and others. Furthermore, Matsudaira *et al*. found that the parental attitude towards praising their child was positively correlated with the GMV in the posterior insula^39^. They suggested that greater GMV in the posterior insula is associated with the recognition of the emotional status of oneself and of others, which facilitates the development of empathy. Based on these two studies, GMV in the posterior insula appears to be associated with cognitions of the self and others, including the emotional processes. However, it is unlikely that our creative insight tasks are related to a cognition of one’s own or other’s emotional processing. Another possibility is that the involvement of the posterior insula is related to motivation due to the dopamine system^40,41^. There is a link between novelty seeking and abundant dopaminergic receptors in the posterior insula^40^. Furthermore, the motivational state with the mean diffusivity measured by diffusion tensor imaging is associated with the motivational state, which is significantly correlated with divergent thinking^41^. We probably also observed a similar mechanism wherein maturation of the posterior insula may contribute to the dopaminergic system, specifically the motivational state.

The ITB score was significantly negatively correlated with the regional GMV of the left CB crus 1 and the right SMA. This result is inconsistent with those of a study that compared art students and non-art students^42^, in which observational drawing ability was positively correlated with GMV in the left anterior CB and the right medial frontal cortex. Chamberlain *et al*. suggested that observational drawing ability relates to changes in GMV structures in the CB to improve motor control and procedural memory. Ben-Soussan *et al*. also reported that structural changes such as GMV and fractional anisotropy in the CB were positively associated with Quadrato Motor Training, which enhances the cognitive flexibility, an important aspect of creativity^43^. In contrast, Taig *et al*. found that patients with cerebellar degeneration were less able to use visual information about the joints for generating motor commands than those without degeneration. Their VBM analysis showed that the inability to use visual information was primarily correlated with degeneration of the CB crus 1. The authors thus suggested that the CB crus 1 plays a key role in the inverse kinematic mapping that enables the use of visual information about body position in the generation of motor commands^44^. Visual information may sometimes disturb the escape from impasse. Limiting the use of visual information may allow representational changes to occur because the subject needs to avoid impasse by using flexible spatial/verbal manipulations to achieve higher creative insight.

We found cerebral-cerebellar networks in the RSFC analysis (insight-positive connections from the CB crus 1 to the orbitofrontal cortex; insight-negative connections from the CB crus 1 to the left IPL). Buckner *et al*. described the cerebral-cerebellar network identified from RSFC data from 1000 subjects^45^. They identified a large region in the CB crus 1 and 2 as a major cerebellar region coupled to the DMN. Another study of improvisation of creative drawing suggested that engagement of the CB, left IPL, right SFC, left PFC and cingulate cortex results in drawings that are more highly rated as creative^13,46^. Individual differences in cerebral-cerebellar networks may therefore represent higher cognitive function, including remembering and planning, which may affect the performance of creative insight.

In the current study, we applied an exploratory approach using a VBM-constrained, seed-based RSFC analysis. By applying this method, Laird *et al*. demonstrated a relationship between working memory and a large-scale network based on multimodal MRI and behavioural data set^47^. The VBM-constrained, seed-based RSFC analysis provided two important advantages over previous research examining RSFC^23,48^. First, we could avoid biased selection of seeds for RSFC analysis. Definition of a seed for RSFC analysis is based on spatial information of a cluster obtained from VBM analysis. Qiu *et al*. used a similar method to identify functional and structural deficits in end-stage renal disease^49^, but most of the previous methods defined a seed based on previous studies, and simply set an ROI (sphere or cube) corresponding to a certain coordinate without consideration of statistical spatial information. We extracted the seed by utilising the cluster information inside grey matter and used AAL2 just to label the clusters. Therefore, we could systematically define the spatial information of the seeds. Second, the VBM-constrained, seed-based RSFC analysis allowed us to extract a cognitive-specific network without prior information or previous knowledge. Some studies have defined a seed’s coordinate in the DMN based on previous studies of creative cognition^7,23^. However, insight-related RSFC has been poorly investigated, so it was difficult to focus on a particular large-scale network with a strong assumption. VBM-constrained RSFC analysis is therefore a useful approach for identification of large-scale networks in the absence of a strong hypothesis or securing spatial information. Of course, there is the limitation that VBM-constrained, seed-based RSFC analysis requires enough data samples, especially when extracting seeds. Sample size should therefore be carefully considered in the design and analysis of such experiments.

We investigated the associations between RSFC and ITB scores, and then illustrated the representation of personal traits for creative insight. However, we did not directly observe brain activity during the ITB task. In a previous study, Jung-Beeman *et al*. designed fMRI and EEG experimental paradigms with verbal problems^14^. One of the most challenging issues for the investigation of creative cognition is detection of the moment when a problem with insight is solved, because creative cognition requires complex cognitive processes and creative cognition tasks require more variations to solve the problem than simple cognitive tasks. Therefore, we decided to first investigate insight-specific large-scale networks as the individual traits of creative insight represented by RSFC. We evaluated the task difficulty before the experiment and excluded participants who had prior experience in solving similar questionnaires to remove training effects. We designed the ITB as a written questionnaire with time limitations (e.g. MA and RAT: 5 minutes; IP: 14 minutes) to efficiently collect behavioural data. However, with this method we could not control the order of the questions to be solved in the test and could not measure the participant response time to find a solution. Therefore, we will directly observe brain activity and its connectivity changes during creative insight processes in the future.

In conclusion, our results suggest that individual differences in creative insight were represented not only by creative-common connections related to divergent thinking and semantic processing, but also an insight-specific network consisting of cerebral-cerebellar networks. These large-scale networks may affect higher cognitive functions and influence the performance of insight problem solving.

## Methods

### Participants

A total of 232 adults aged 21 to 69 years old participated in this study. We recruited participants as two independent cohorts (group 01: 101 participants; group 02: 131 participants). Participants received cash payment and a postcard for their involvement in this study. All participants had normal or corrected-to-normal vision and no reported history of drugs that affect the CNS, and no neurological disease or diabetes. All participants provided written informed consent prior to the experiment. This study was approved by the ethical committee of the Advanced Telecommunication Research Institute International (ATR) and followed the Declaration of Helsinki.

### Experimental procedure

Each participant completed the MRI and cognitive tasks over two days. MRI data acquisition and a working memory task were completed on Day 1, and the ITB and two cognitive tasks (the sequential fixed index task and the dot probe task) were completed on Day 2. After Day 1, the participants performed assessments of cognition and personality in a self-paced manner.

Insight for creative cognition was evaluated using the ITB, which consisted of three tasks (the MA^25^, RAT^26^ and IP^27^). All tasks were hand-written on test papers (an example is shown in Supp. Fig. 1). The test periods for MA, RAT and IP were 5 min, 5 min and 14 min, respectively. We evaluated the relationship between MA, RAT and IP scores using partial correlation analysis (‘partialcorr.m’ in the Statistics and Machine Learning Toolbox of MATLAB) after controlling for age, sex and cohort group.

### MRI Data acquisition

Images were acquired with 3 T MRI scanners, MAGNETOM Trio Tim (Trio), MAGNETOM Verio (Verio), and MAGNETOM Prisma or Verio (Siemens Medical Systems, Erlangen, Germany) installed in the Brain Activity Imaging Center (BAIC) in the ATR. The MRI of Trio was upgraded between group 01 and 02, so we used Trio and Verio for group 01 and Prisma and Verio for group 02. High-resolution T1-weighted structural images were acquired for normalisation to a standard brain for echo planar image (EPI) registration purposes and the VBM analysis (TR = 2300 ms, TE = 2.98 ms, flip angle = 9 degrees, TI = 900 ms, matrix = 256 × 256, field of view = 256 mm, slice thickness = 1 mm, iso-voxel). Functional images were acquired with an EPI sequence (TR = 2500 ms, TE = 30 ms, flip angle = 80 degrees, matrix = 64 × 64, field of view = 212 mm, slice thickness = 3.2 mm, gap: 0.8 mm, 40 slices (Trio/Prisma) or 39 slices (Verio), scan sequences: ascending) of 244 volumes at rest for 10 min. During the resting-state scan, the participants were instructed to keep looking at a central fixation point, to keep still, to stay awake, and not to think about specific things.

### Voxel-Based Morphometry analysis

Preprocessing of the morphological data was performed using VBM-DARTEL in SPM12 (Wellcome Trust Centre for Neuroimaging) and default parameter settings were used. Participant numbers for each age range and sex are described in Table 1. First, 162 individual MR images were displayed in SPM12 to screen out artefacts or gross anatomical abnormalities, excluding the 70 participants who had prior experience with MA or IP tasks. To reduce scanner-specific bias, the reorientation of the images was semi-automatically set to the anterior commissure. The images were segmented into grey matter, white matter and cerebrospinal fluid (CSF) by using “old segment” before starting segmentation in SPM12 to adjust background noise. We performed registration, then normalisation and modulation using DARTEL. To ensure that regional differences in the absolute amount of grey matter were conserved, each voxel was modulated by Jacobian determinants derived from spatial normalisation. Subsequently, registered images were transformed to the MNI space. Finally, the modulated images (grey matter) were smoothed with an 8-mm full-width at half-maximum Gaussian kernel to increase the signal-to-noise ratio.

The group analysis of the GMV data was performed with VBM in SPM12. At the group level analysis, we tested 162 images for the relationship between the ITB score (sum of normalised MA, RAT and IP scores) and GMV. We used multiple linear regression analysis to look for regions in which the GMV showed a significant relationship with the ITB score. The level of statistical significance was set at *p* < 0.001 (uncorrected). The analysis included age, sex (male/female), MRI scanner (Trio/other, Prisma/other), cohort (group 01/group 02) and TBV. To avoid edge effects around the borders between grey matter and white matter, we used explicit masking to restrict the search volume. From 162 grey matter segmented images, explicit masking was achieved by masking a toolbox in SPM12 (http://www0.cs.ucl.ac.uk/staff/g.ridgway/masking). This approach reduced the risk of false negatives caused by overly restrictive masking, which decrease the possibility that voxels of interest are excluded from the statistical analysis^50^.

### Resting-state fMRI data preprocessing

The data were processed using SPM12. The first four volumes were discarded to allow for T1 equilibration. The remaining data were corrected for slice timing and realigned to the mean image of that sequence to compensate for head motion. Next, the structural image was coregistered to the mean functional image and segmented into three tissue classes in the MNI space. Using associated parameters, the functional images were normalised and resampled in a 2 × 2 × 2 mm grid. Finally, they were spatially smoothed using an isotropic Gaussian kernel of 8 mm full-width at half maximum.

### VBM constrained seed-based RSFC analysis

For each participant, a correlation map was produced by extracting the averaged fMRI time course within a seed region that was obtained from the VBM analysis. Next, we computed the correlation coefficient between the seed’s time course and time courses from each other brain voxel (*r*-map). We calculated the *z-*map using Fisher’s z-transformation to improve normality of the *r*-map. To remove several sources of spurious variance along with their temporal derivatives, linear regression was performed, including six motion parameters in addition to averaged signal over grey matter, white matter and CSF. Furthermore, to reduce spurious changes in functional connectivity by head motion, the data were checked by a method that reduces motion-related artefacts. Specifically, we calculated frame-wise displacement (FD) and DVARS (D: temporal derivative of time-courses, VAR: root mean square variance over voxels) and removed volumes with an FD > 0.5 mm or DVARS > 0.5%, as proposed by a previous article^28^. A band-pass filter (transmission range, 0.008–0.1 Hz) was applied to these sets of time courses prior to the following regression procedure. Nineteen participants were excluded from the RSFC analysis because the number of excluded volumes was more than 20% of the total volume.

In the second-level analysis, we examined the relationship between changes in the ITB score and changes in the RSFC across the participants. To investigate these relationships in the whole brain analysis, we used multiple linear regressions using SPM12 to search for areas where RSFC from a seed was significantly associated with the ITB score. A pair-wise temporal correlation between the mean time course of each seed region and all voxels in the grey matter were first computed for each participant. An individual level RSFC correlation map was then produced within the whole brain. To model the relationship between changes in RSFC and changes in the ITB score, we assumed the general linear model to explain the RSFC estimated by covariates (age, sex, ITB score, MRI [Trio], MRI [Prisma] and cohort [group 01/02]) for all voxels across the participants. The initial voxel threshold was set to *p* < 0.001 uncorrected for multiple comparisons. Clusters were considered significant if they survived an extent threshold of *p* = 0.1 or 0.05 Family-Wise Error corrected for multiple comparisons for statistical inference^51^. A cluster consisting of a positive/negative T score was defined as a “task-positive” connection or “task-negative” connection, respectively. The brain networks were visualised with BrainNet Viewer (http://www.nitrc.org/projects/bnv)^52^. Volumes of clusters, shown in Table 4, were obtained from anatomical region of interests labelled by the AAL2 toolbox^53^.

### Data availability

The datasets of 192 participants analysed during the current study are partly available in the ATR Wide-Age-Range Brain Data Repository (https://bicr-resource.atr.jp/impact/).

## Electronic supplementary material

Supplemental information
