# Supplementary material for: Large-scale brain network associated with creative insight: combined voxel-based morphometry and resting-state functional connectivity analyses
Source: Sci Rep. 2018 Apr 24;8:6477. doi: 10.1038/s41598-018-24981-0 (PMC5915578; doi:10.1038/s41598-018-24981-0)
Supplement: Supplementary file 1 — Supplemental information [file 41598_2018_24981_MOESM1_ESM.docx]

# Supplemental information

**Title: Large-scale brain network associated with creative insight: combined voxel-based morphometry and resting-state functional connectivity analyses**

**Authors and affliations:**

Takeshi Ogawa^1*^, Takatsugu Aihara^2^, Takeaki Shimokawa^2^ and Okito Yamashita^2^

^1^ATR Cognitive Mechanisms Laboratories, Kyoto 619-0288, Japan

^2^ATR Neural Information Analysis Laboratories, Kyoto 619-0288, Japan

* Corresponding author:

Takeshi Ogawa, Ph D. in Medicine

Address: 2-2-2, Hikaridai, Seika-cho, Soraku-gun, Kyoto, 619-0288, Japan

Tel: +81-774-1413

Fax: +81-774-1236

E-mail: t.ogawa@atr.jp

## **Supplementary Figure 1**: Examples of the insight task.

(A) Matchstick arithmetic task


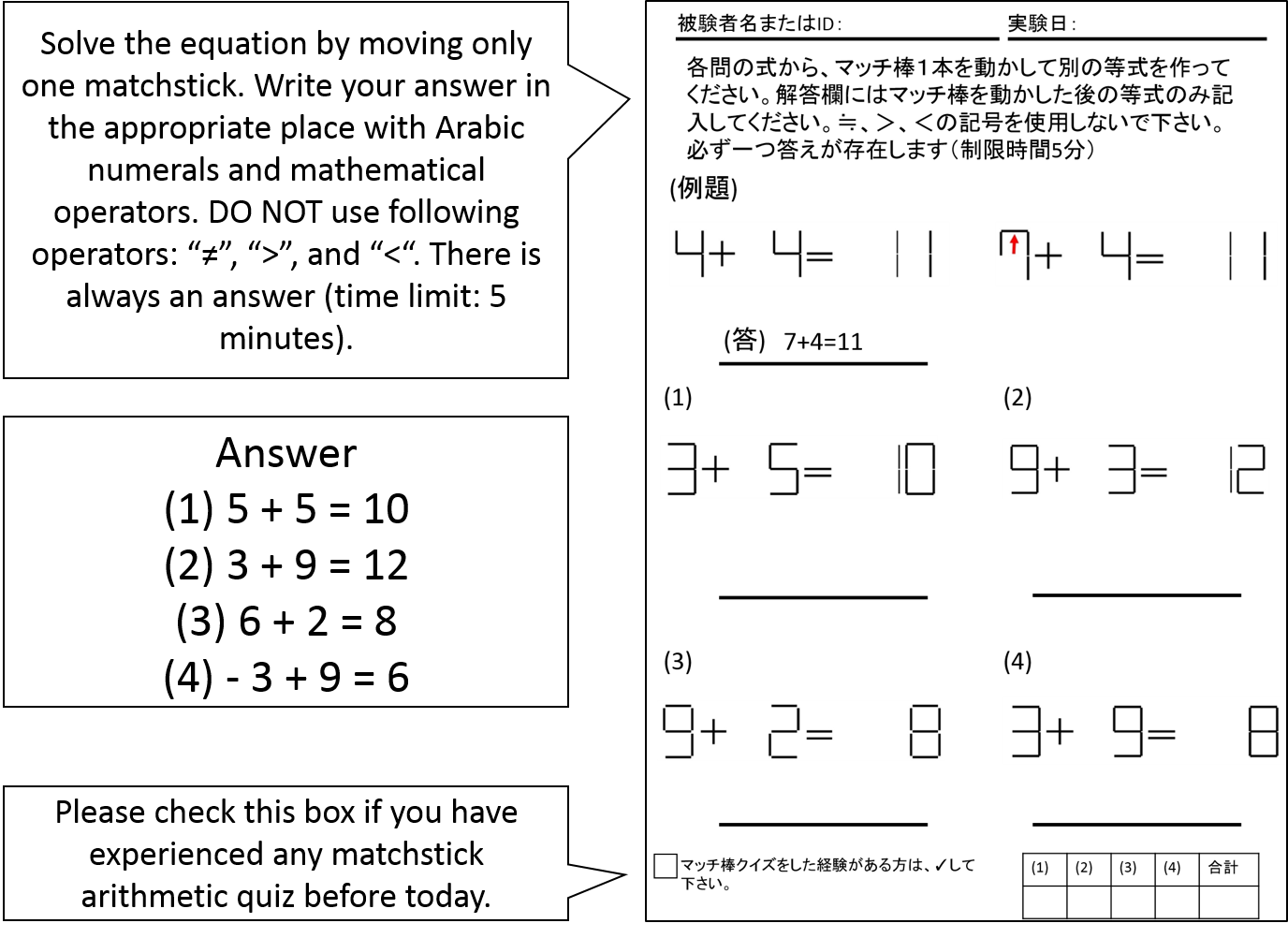


(B) Remote Associates Test


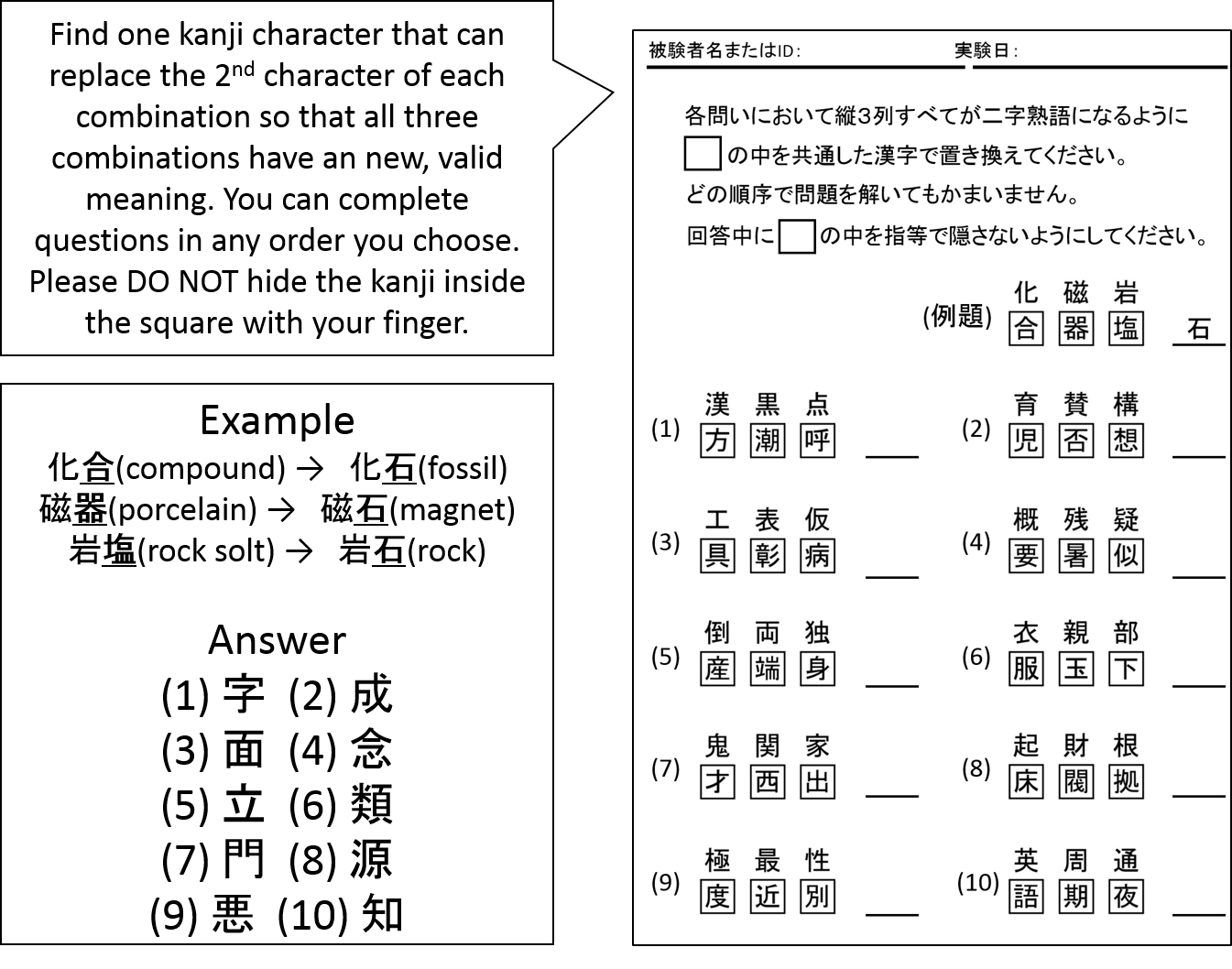


(C) Insight Problem task


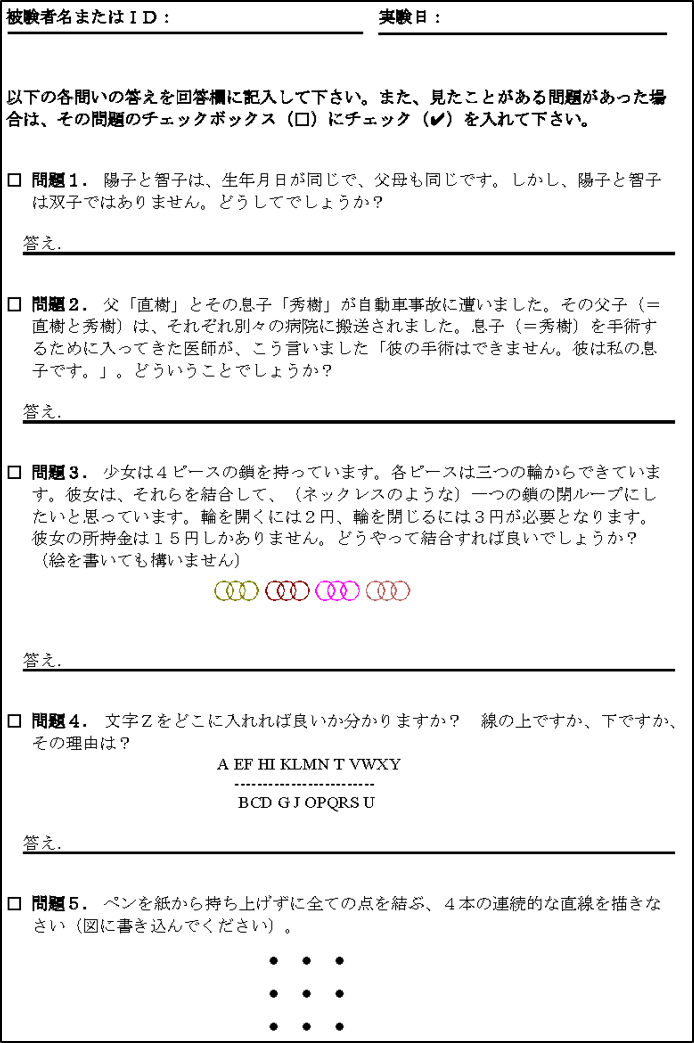


Supplementary Figure1: Examples of the insight task.

The MA task (A) measures the processes of chunk decomposition and constraint relaxation (Ollinger et al., 2008). In this task, an incorrect/correct initial equation, represented by matchsticks, is given in Arabic numerals; the participant must solve the equation by moving only one matchstick. We set three Type I questions (which require a simple chunk to be decomposed, i.e. a matchstick is moved to/from a number) and one Type II question (which requires a more difficult chunk to be decomposed, i.e. a matchstick is moved from a numeral to an arithmetic operator). The RAT (B) measures creative ability, particularly, convergent thinking based on chunk decomposition. Bowden and Jung-Beeman (2003) developed the RAT as an insight problem-solving task. Terai et al. (2013) developed a Japanese version of RAT using Japanese Kanji characters. One word consisted of two kanji, and three different words were presented. The bottom kanji in the square was a distractor. Participants were required to answer a common kanji for three words to create another correct three words. We selected 10 questions (5 rows x 2 columns) from Terai et al.’s RAT (2013). The IP (C) measures the skill of insight problem solving. The IP we used consisted of the two categories: verbal and spatial (<http://www.indiana.edu/~bobweb/insightproblems.doc>; Dow and Mayer, 2004). We chose five problems (verbal insight problems: #3 (twins), #35 (car accident); spatial insight problems: #3 (chain), #11 (Letter Z), #13 (9 dots)) and translated them into Japanese. Participants were asked to provide reasons or draw graphs. The IP was scored following the answers of the original version. Ambiguous answers were judged by majority vote of five examiners.

Abbreviations: IP: insight problem task; MA: matchstick arithmetic task; RAT: remote associates test.

## **Supplementary Table 1**: Correlations between individual RSFCs and each task score at the coordinate of the peak voxel.

| Region | | ITB | | IP | MA | RAT |
| --- | --- | --- | --- | --- | --- | --- |
| Seed 2: R MCC | | | | | | |
| R MTG | | 0.35 (1e-5) | | 0.32 (7e-5) | 0.16 (0.05) | 0.22 (0.01) |
| Seed 3: CB crus 1 | | | | | | |
| R Med Orb Frontal | 0.38 (2e-6) | | 0.30 (3e-4) | | 0.20 (0.02) | 0.27 (1e-3) |
| L IPL | -0.38 (2e-6) | | -0.26 (1e-3) | | -0.24 (4e-3) | -0.26 (1e-3) |

Abbreviations: CB: cerebellum; IP: insight problem task; IPL: inferior parietal lobule; ITB: insight test battery; L: left; MA: matchstick arithmetic task; MCC: middle cingulate cortex; Med Orb Frontal: medial orbitofrontal; R: right; RAT: remote associates test.

# References

Bowden, E. M. and Jung-Beeman, M. Aha! Insight experience correlates with solution activation in the right hemisphere. *Psychon Bull Rev.* 10, 730-737 (2003).

Dow, G. T. & Mayer, R. E. Teaching students to solve insight problems. Evidence for domain specificity in training. *Creativity Res J.* 16, 389- 402 (2004).

Ollinger, M., Jones, G. & Knoblich, G. Investigating the effect of mental set on insight problem solving. *Exp Psychol.* 55, 269-282 (2008).

Terai H., Miwa, K. & Asami, K. Development and evaluation of the Japanese remote associates test. *Shinrigaku Kenkyu* **84**, 419-428 (2013).
